# Effects of dexmedetomidine on evoked potentials in spinal surgery under combined intravenous inhalation anesthesia: a randomized controlled trial

**DOI:** 10.1186/s12871-023-01990-9

**Published:** 2023-01-31

**Authors:** Xinyu Jiang, Xiaoning Tang, Shaoquan Liu, Ling Liu

**Affiliations:** 1 Department of Anesthesiology, The First Affiliated Hospital of Chongqing Medical University, No.1 Youyi Road, Yuzhong District Chongqing, 400016 People’s Republic of China; 2 Department of Orthopedics, The First Affiliated Hospital of Chongqing Medical University, Chongqing, 400016 People’s Republic of China

**Keywords:** Dexmedetomidine, Evoked potentials, Spinal surgery

## Abstract

**Objective:**

We aimed to investigate the effects of different doses of dexmedetomidine (Dex) on evoked potentials in adult patients undergoing spinal surgery under intravenous anesthesia with low-concentration desflurane.

**Methods:**

Ninety patients were divided into three groups at random. To maintain anesthesia in the control group (group C), desflurane 0.3 MAC (minimal alveolar concentration), propofol, and remifentanil were administered. Dex (0.5 μg·kg^−1^) was injected for 10 min as a loading dose in the low-dose Dex group (group DL), then adjusted to 0.2 μg·kg^−1^·h^−1^ until the operation was completed. Dex (1 μg·kg^−1^) was injected for 10 min as a loading dose in the high-dose Dex group (group DH), then adjusted to 0.7 μg·kg^−1^·h^−1^ until the operation was completed. The additional medications were similar to those given to group C. The perioperative hemodynamics, body temperature, intraoperative drug dosages, fluid volume, urine volume, blood loss, the latency and amplitude of somatosensory evoked potentials (SEPs) at four different time points, the incidence of positive cases of SEPs and transcranial motor evoked potentials (tcMEPs), and perioperative adverse reactions were all recorded.

**Results:**

Data from 79 patients were analyzed. The MAP measured at points T2-T4 in group DH was higher than at corresponding points in group C (*P* < 0.05). The MAP at point T4 in group DL was higher than at corresponding points in group C (*P* < 0.05). The remifentanil dosage in group DH was significantly lower than in group C (*P* = 0.015). The fluid volume in group DL was significantly lower than in group C (*P* = 0.009). There were no significant differences among the three groups in the amplitude and latency of SEP at different time points, nor in the incidence of warning SEP signals. The incidence of positive tcMEP signals did not differ significantly between groups C and DL (*P* > 0.05), but was significantly higher in group DH than in groups DL (*P* < 0.05) or C (*P* < 0.05). The incidence of intraoperative hypertension was significantly higher in group DH than in group C (*P* = 0.017).

**Conclusions:**

Low-dose Dex has no effect on the SEPs and tcMEPs monitoring during spinal surgery. High-dose Dex has no effect on SEPs monitoring, but it may increase the rate of false positive tcMEPs signals and the incidence of intraoperative hypertension.

**Trial registration:**

This study has completed the registration of the Chinese Clinical Trial Center at 11/09/2020 with the registration number ChiCTR2000038154.

## Introduction

A catastrophic complication of spinal surgery is nerve and spinal cord injury [1]. The incidence of neurological defects after spinal surgery can be reduced from 3.7%-6.9% to less than 1% with proper electrophysiological monitoring [2]. Somatosensory evoked potentials (SEPs) and transcranial motor evoked potentials (tcMEPs) are currently used as adjunct diagnostic methods in spinal surgery, such as scoliosis surgery and spinal stenosis decompression. Total intravenous anesthesia (TIVA) with propofol and opioids is commonly used in SEPs and tcMEPs monitoring [3]. Long-term use of propofol at high doses, on the other hand, can result in complications such as delayed awakening, hypertriglyceridemia, and abnormal platelet function [4]. Furthermore, long-term propofol infusion can cause a significant decrease in tcMEPs amplitude, a phenomenon known as anesthetic fade [5]. The amplitudes of tcMEPs and SEPs are reduced by halogenated volatile anesthetics, limiting their use in spinal surgery that requires electrophysiological monitoring [6, 7]. When volatile anesthetics did not exceed 0.3MAC, they had little effect on tcMEPs and SEPs [7, 8]. Martin et al. discovered that volatile agent-based anesthesia has application value during neurophysiological monitoring, such as faster awakening and rapid wake-up tests [2]. Simultaneously, volatile anesthetics can reduce the dosage of propofol. As a result, spinal surgery benefits from combined intravenous inhalation anesthesia. As an adjuvant, dexmedetomidine (Dex) may be useful in reducing the need for propofol [9, 10]. For example, the induction dose of propofol can be reduced by 15% and the demand for propofol can be reduced by 29% by adding Dex to TIVA [11]. Dex allows patients to maintain non-rapid eye movement sleep that can be stimulated or awakened by language [12], which is useful for spinal surgery where an arousal test is required. Simultaneously, Dex may protect against nerve ischemia–reperfusion injury and reduces the incidence of intraoperative and postoperative adverse reactions [13, 14], providing the theoretical basis for including Dex in the spinal surgery anesthetic scheme. However, many trials were designed based on intravenous anesthesia and a single dose of Dex [15, 16]. It is worth investigating whether dexmedetomidine will exacerbate the effects of combined intravenous inhalation anesthesia on tcMEPs and SEPs. The purpose of this study was to see how different doses of Dex affected evoked potentials in patients undergoing intravenous anesthesia with low-concentration desflurane.

## Methods

This single-center, randomized study was carried out in the First Affiliated Hospital of Chongqing Medical University. The study was approved by the First Affiliated Hospital of Chongqing Medical University (reference number was 2020–390), and written informed consent was obtained from all individual participants before surgery for data collection and analysis.

### Patients and groups

**Fig. 1:**
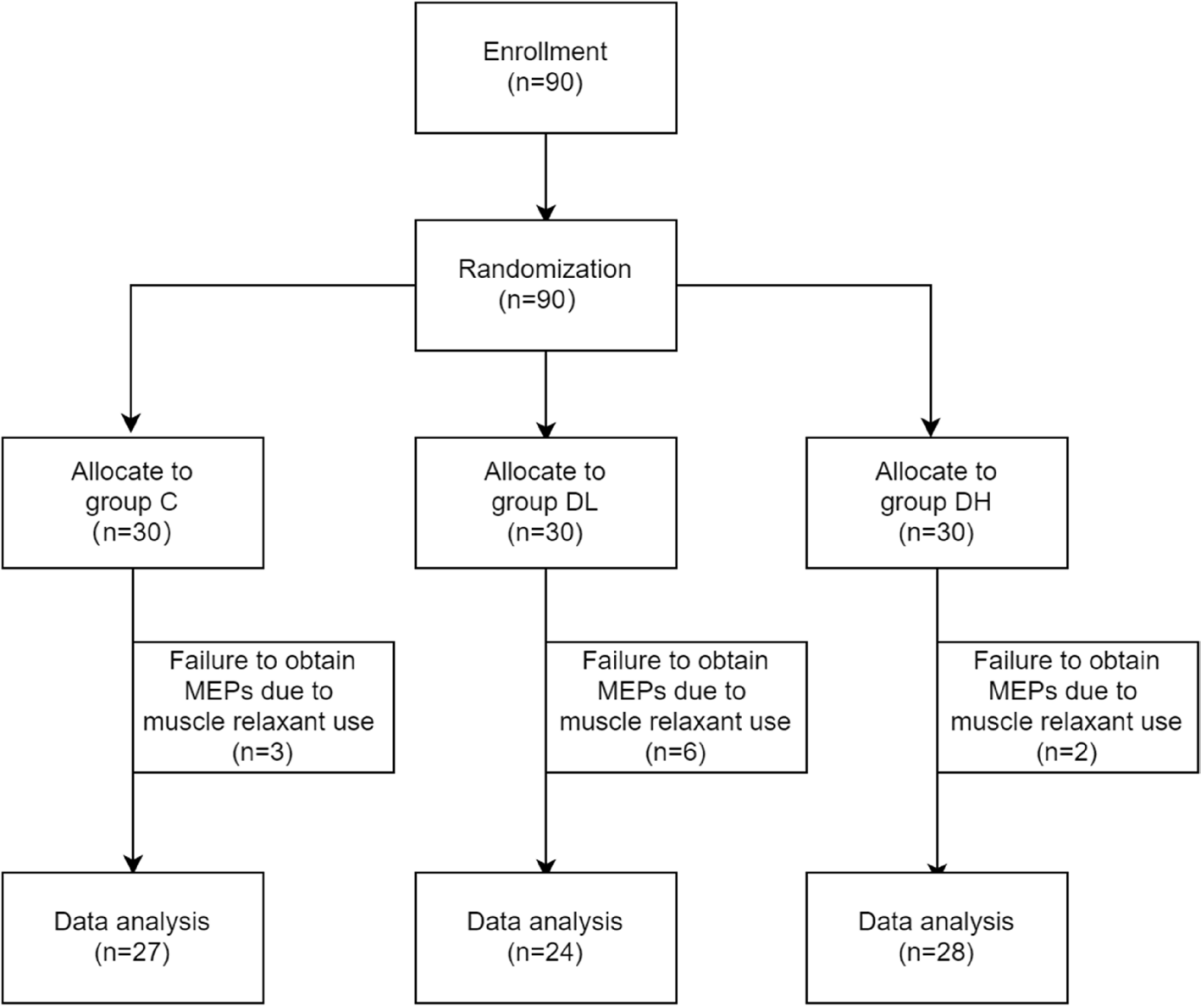
The flow chart of patient selection

Patients undergoing spinal surgery who required electrophysiological monitoring were included in this study. Other inclusion criteria included American Society of Anesthesiologists (ASA) physical status I-III, muscle strength III-V (Lovett muscle strength grading), and age between 18 and 80 years old. Excluded from the study were patients with nerve conduction pathway injury, intracranial hypertension syndrome, diabetic peripheral neuropathy, myasthenia gravis, obvious abnormal liver and kidney function, severe circulatory respiratory system disease, mental disorder, pacemaker implantation, history of epilepsy, skull defect, auditory impairment, and language communication difficulties. Patients who needed to be awakened during the procedure were also excluded. The flow chart of patient selection was shown in Fig. 1.

Patients were randomly assigned to one of three groups (*n* = 30 each): group DL (0.5 μg·kg^−1^ loading dose Dex infused for 10 min followed by a constant infusion rate of 0.2 μg·kg^−1^·h^−1^), group DH (1.0 μg·kg^−1^ loading dose Dex infused for 10 min followed by a constant infusion rate of 0.7 μg·kg^−1^·h^−1^), and group C. Dex doses were determined using medication instructions of dexmedetomidine (Dexmedetomidine Hydrochloride Injection, dexmedetomidine hydrochloride, Yangtze River Pharmaceutical, Taizhou City, Jiangsu Province, China) and expert consensus on the clinical application of Dex. SPSS software (Version 21.0, SPSS) random number generator was used to generate random sequences, and covert grouping was done with continuously coded, sealed, opaque envelopes. Because the anesthesiologist was required to manage anesthesia, neurophysiologists and patients were subjected to blind methods until the procedure was completed.

### Anesthesia methods

#### Anesthesia induction

Patients were required to fast for 8 h before the procedure. Both the electrocardiogram (ECG) and the finger pulse oxygen saturation were monitored, and intravenous access was performed on a regular basis. To monitor blood pressure and collect blood samples, radial artery cannulation was performed under local anesthesia. The three groups used the same method for anesthesia induction. Anesthesia was administered via intravenous rapid induction to each group. Propofol 1.5–2 mg·kg^−1^, sufentanil 0.3–0.5 μg·kg^−1^, midazolam 0.05 mg·kg^−1^, and rocuronium 0.6 mg·kg^−1^ were used to induce anesthesia. Intraoperative muscle relaxants were no longer used (cases who received additional muscle relaxants were excluded). After 5 min of oxygen and denitrification, endotracheal intubation was performed, and mechanical ventilation was connected to the ventilator. The rate of respiration was adjusted to keep the end-tidal carbon dioxide between 30 and 35 mmHg.

#### Anesthesia maintenance

The Narcotrend (MT MonitorTechnik GmbH&Co.KG, Germany) was used in all cases to monitor the depth of sedation, with the stage maintained at D0-D2. In group C, 0.3 MAC desflurane, propofol 4–12 mg·kg^−1^·h^−1^, and remifentanil 0.05–0.3 μg·kg^−1^·min^−1^ were used to maintain anesthesia. Except for Dex, the drugs used in groups DL and DH were identical to those used in group C. Train-of-four (TOF) stimulation modes were used to monitor muscle relaxation. The dexmedetomidine loading dose was started when T4/T1 > 75% and the baseline signal was obtained. If T4/T1 did not reach 75% 30 min after induction, neostigmine (0.05–0.07 mg·kg^−1^) and atropine were used to reverse the muscle relaxation effect of non-depolarizing muscle relaxants. The mean arterial pressure (MAP) was kept within a range of 60 to 85 mmHg. Small doses of vasoactive drugs (such as ephedrine 5–15 mg or norepinephrine 50–200 ug) could be used if the MAP was consistently lower than 60 mmHg (> 1 min) and difficult to maintain by adjusting the infusion rate of anesthetics and rehydration. A low dose of urapidil 5–10 mg was used if the MAP was consistently greater than 85 mmHg and there was no shallow anesthesia or carbon dioxide accumulation. 0.25–0.5 mg atropine could be used if the heart rate (HR) was less than 40 beats per minute. The body temperature was kept at 36 ± 1 ℃.

### Recordings of the tcMEPs and SEPs

A 32-channel Cadwell Cascade neurophysiological monitoring system (Cadwell Laboratories, Kennewick, WA) was used for stimulation and recording. The scalp electrodes were positioned in accordance with the international 10–20 montage system.

SEPs were obtained continuously by stimulating the upper and lower extremities during the operation. Patch electrodes were used to stimulate the median nerve, ulnar nerve, and posterior tibial nerve at the wrist and ankle, respectively. The anode was at the distal end, and the cathode was at the proximal end. Recording electrodes were placed on Cz (both lower limbs), C3', C4' (both upper limbs). Stimulus parameters were set as follows: intensity 15-25 mA, stimulus interval 100-300 ms, and stimulus frequency 2.1–4.7 Hz. The following recording parameters were used: the filter bandwidth was set to 30-600 Hz, the 50 Hz notch was turned off by default, the average number of signals was set to 100–400, and the analysis time was set to 50 ms.

TcMEPs were performed with spiral electrodes to stimulate the C3, C4 or C1, C2 region (C1 is 10% to the left of Cz, C2 is 10% to the right of Cz) with eight pulses (biphasic square pulses). According to different surgical requirements, compound muscle action potentials were recorded by straight needle electrodes on deltoid muscle, biceps brachii muscle, extensor carpi longus muscle, abductor pollicis brevis muscle and abductor digitorum minatus muscle of upper limb, quadriceps femoris muscle, tibialis anterior muscle, extensor pollicis longus muscle, abductor pollicis muscle and calf muscle of lower limb. The stimulus parameters were as follows: stimulus intensity ranged from 100 to 300 V, interval time was set to 2 ms, and stimulus interval was set to 0.1 ms. Recording parameters were set as follows: the filter bandwidth was set to 30-2500 Hz, the 50 Hz notch was turned off by default, the average number of signals was one, and the signal analysis time was 100 ms. The current's power and intensity were set at the outset and maintained throughout the procedure.

During the operation, SEPs were routinely recorded every 10 min, and tcMEPs were recorded every 10 min (not at the same time). Multiple somatosensory and motor waveforms were recorded if the surgeon needed to record or monitor the abnormal state. The recordings were made in the following order: first, motor stimulation (left and right), then somatosensory stimulation. The order was left upper limb, right upper limb, left lower limb, and right lower limb.

When the monitoring signal(s) of SEPs or (and) MEPs reached the warning value, the surgeons were notified and the operation was temporarily stopped. If the waveform did not recover, the Dex infusion was terminated and a wake-up test was carried out. The true positive case referred to an electrophysiological warning signal and postoperative neural injury. The false-positive case referred to an electrophysiological warning signal without postoperative neural injury. The false-negative case referred to a normal electrophysiological signal but postoperative neural injury. The true negative case referred to a normal electrophysiological signal with no postoperative neural injury.

### Data acquisition

Baseline patient characteristics (age, gender, body mass index (BMI), ASA physical status, muscle strength), operation time, blood loss, fluid replacement, urine volume, and body temperature were all recorded. T0 was before anesthesia induction, T1 was after anesthesia induction and before Dex infusion, T2 was 10 min after Dex administration, T3 was 20 min after Dex administration, and T4 was 30 min after Dex administration (T4). The HR and MAP were recorded at T0, T1, T2, T3, and T4. The primary endpoint was the incidence of positive cases of tcMEPs. The secondary endpoints included the latency and amplitude of SEPs at T1, T2, T3, T4, and the incidence of positive cases of SEPs, the propofol and remifentanil dosages, the occurrence of perioperative adverse reactions.

### Statistical analysis

The sample size was calculated using the PASS11 software for Windows 10. According to the results of Lee [15], the tcMEPs amplitude (500 µV; SD,110) was used to detect a 30% difference in tcMEPs amplitude (from 500 to 350 µV) in the bilateral test when the alpha value was 0.01 and the study power was 0.90. The sample size for each group was calculated to be 26 based on early termination and a 10% withdrawal rate during the study period. Our experiment included 30 subjects in each group.

Statistical analyses were carried out using the Statistical Package for Social Sciences (version 21.0, SPSS). All quantitative variables were examined for normal distribution and variance homogeneity. Data with a normal distribution were expressed as the mean ± standard deviation (mean ± SD), and one-way analysis of variance (ANOVA) was used to compare differences between groups. The non-normal distribution data were presented as median (first quartile, third quartile), and the group differences were compared using the non-parametric Kruskal–Wallis test. The qualitative variables were presented as numbers/percentages, and the groups were compared using the Pearson's chi-squared test or the Fisher exact test. All *P* values were two-sided, and the threshold for statistical significance was set at *P* < 0.05.

## Results

The study included 90 patients who underwent spinal surgery between September 11, 2020 and September 12, 2021. 11 patients were excluded from the analysis because the surgeon required additional muscle relaxants due to muscle tension during the operation, preventing the continuous measured tcMEPs signal from being obtained (3, 6, 2 in 3 groups respectively).

### Comparison of general and intraoperative conditions among 3 groups of patients

There were no significant differences in age, gender, BMI, operation time, or anesthesia time among the three groups (*P* > 0.05), as shown in Table 1.

**Table 1:** Demographic data(mean ± SD)

Group	Age (years)	Gender (male,%)		BMI	Operation time (minutes)	Anesthesia time (minutes)	Surgery (cervical/thoracic/lumbar vertebra)
C(*n* = 27) DL(*n* = 24) DH(*n* = 28)	57 ± 12 58 ± 14 52 ± 15	12,44 10,42 14,50	24 ± 3 24 ± 3 23 ± 3		163 ± 73 139 ± 57 133 ± 42	203 ± 76 178 ± 60 171 ± 46	13/4/10
							10/3/11
							7/2/19
P	0.313	0.826	0.736		0.150	0.148	

*P* > 0.05, no significant difference in the three groups. One-way analysis of variance for multiple groups of continuous variables

*Abbreviation:* *BMI* body mass index

### Comparison of MAP, HR and core temperature among 3 groups

At T0 and T1, there were no significant differences in MAP between groups (*P* > 0.05). The MAP measured at points T2-T4 in group DH, on the other hand, was higher than at corresponding points in group C (*P* < 0.05). Furthermore, the MAP at T4 in group DL was higher than at the same point in group C (*P* < 0.05). There were no significant differences in HR or core temperature between the three groups at different points of time (*P* > 0.05). See Table 2 for more information.

**Table 2:** Hemodynamic data and core temperature at different points of time (mean ± SD)

Variable	Group	T0	T1	T2	T3	T4
MAP (mmHg)	C(*n* = 27)	93 ± 8	81 ± 8	79 ± 5	80 ± 6	79 ± 6
	DL(*n* = 24)	99 ± 11	84 ± 11	85 ± 10	84 ± 11	86 ± 10^*^
	DH(*n* = 28)	92 ± 11	81 ± 7	89 ± 15^*^	89 ± 12^*^	89 ± 11^*^
	P	0.055	0.366	0.005	0.004	< 0.001
HR (bpm)	C(n = 27)	72 ± 10	63 ± 11	62 ± 10	62 ± 6	60 ± 6
	DL(*n* = 24)	76 ± 12	66 ± 9	61 ± 7	60 ± 7	61 ± 7
	DH(*n* = 28)	76 ± 10	62 ± 9	60 ± 10	62 ± 11	64 ± 9
	P	0.301	0.470	0.666	0.737	0.260
Core temperature (℃)	C(*n* = 27)	36.4 ± 0.28	36.4 ± 0.28	36.4 ± 0.24	36.4 ± 0.26	36.3 ± 0.26
	DL(*n* = 24)	36.5 ± 0.34	36.6 ± 0.37	36.6 ± 0.40	36.6 ± 0.39	36.5 ± 0.38
	DH(*n* = 28)	36.5 ± 0.28	36.5 ± 0.25	36.5 ± 0.22	36.5 ± 0.19	36.5 ± 0.24
	P	0.205	0.059	0.127	0.095	0.063

*Abbreviations:* *MAP* mean arterial pressure, *HR* heart rate

^*^P < 0.05, compared with group C. One-way analysis of variance for multiple groups of continuous variables

### Comparison of medications dosage during operation among 3 groups

**Fig. 2:**
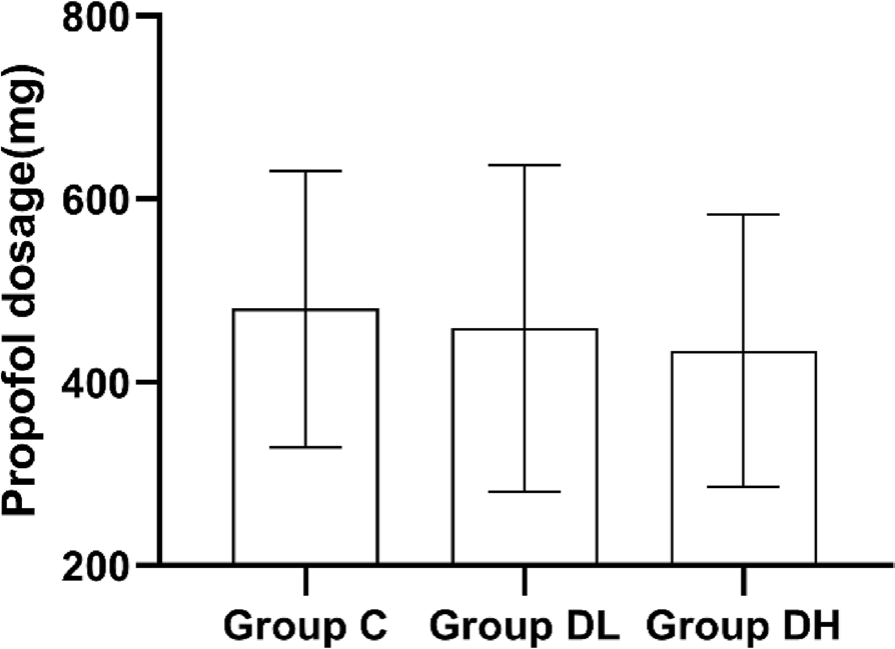
The dose of intraoperative propofol

**Fig. 3:**
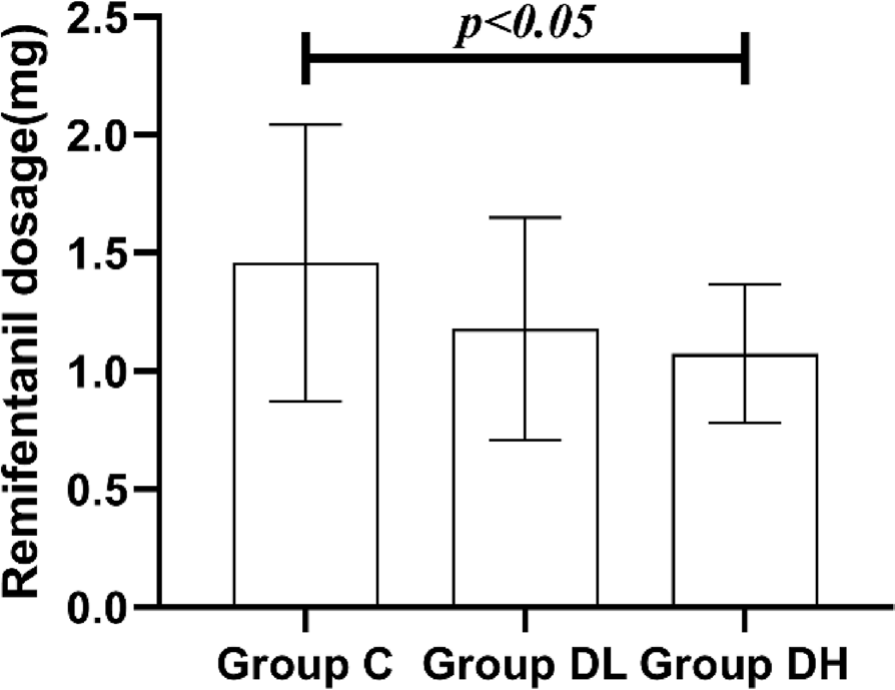
The dose of intraoperative remifentanil

The dosage of propofol did not differ significantly between the three groups (*P* = 0.576). However, the remifentanil dosage was lower in group DH than in group C (*P* = 0.015). See Figs. 2 and 3.

### Comparison of fluid volume, urine volume, and blood loss among 3 groups

**Fig. 4:**
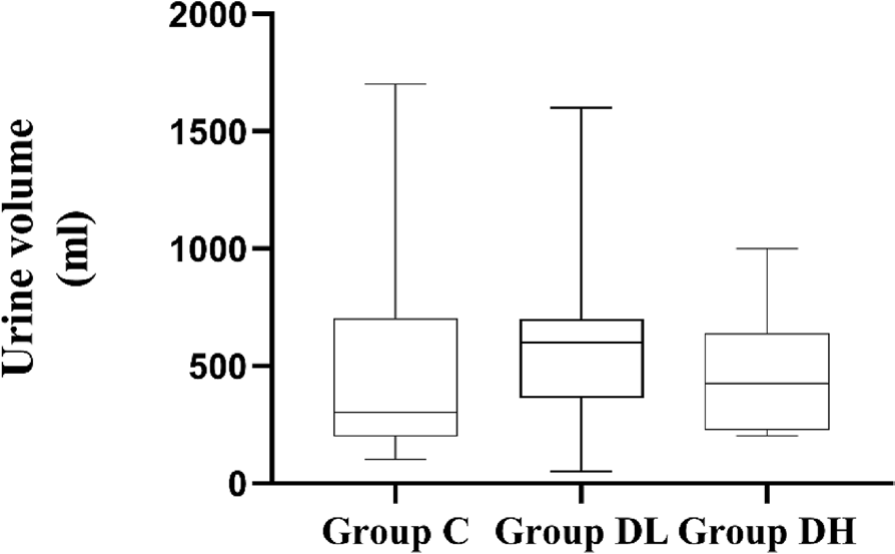
The urine volume during the procedure

**Fig. 5:**
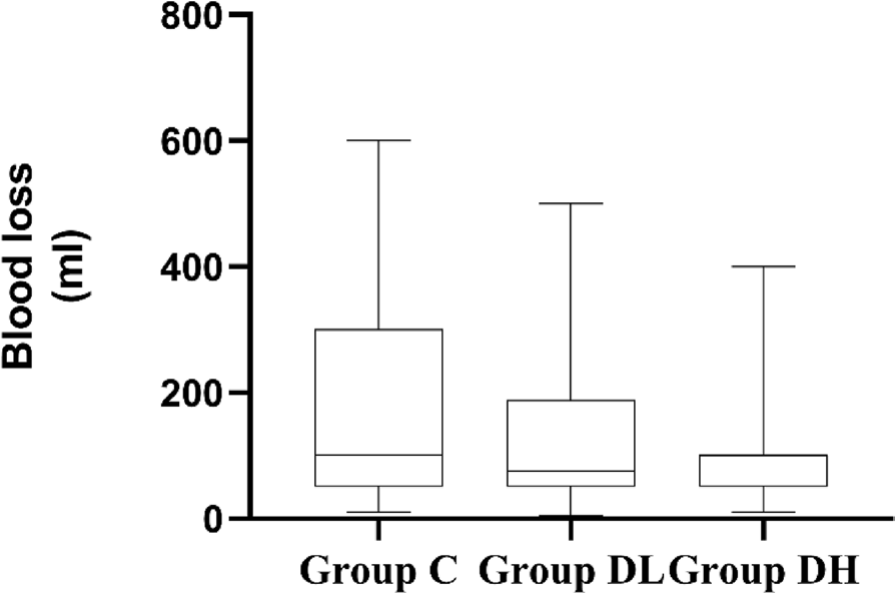
The blood loss during the procedure

**Fig. 6:**
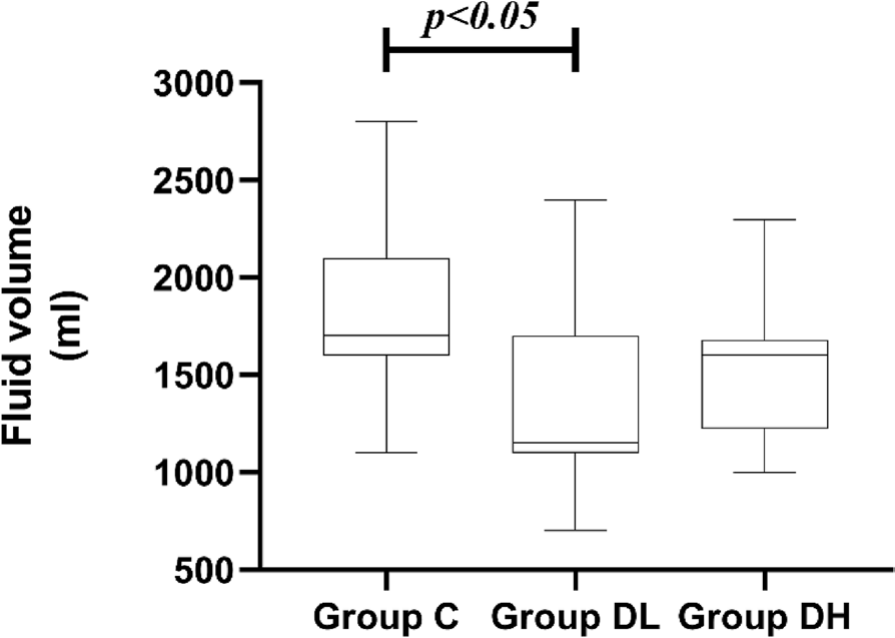
The fluid volume during the procedure

There were no statistically significant differences in urine volume or blood loss between the three groups (*P* > 0.05). Fluid volume was significantly lower in group DL than in group C (*P* = 0.009). See Figs. 4, 5 and 6.

### Comparison of the latency and the amplitude of SEPs (P40-N45) at different time points

There were no significant differences in the amplitude or latency of SEPs (P40-N45) at different points of time between the three groups (*P* > 0.05). See Tables 3 and 4.

**Table 3:** The latency of SEPs (P40-N45) at different points of time (mean ± SD)

	Group	T1	T2	T3	T4
Latency of left lower limb (ms)	C(*n* = 27)	41.1 ± 2.9	41.1 ± 2.6	41.3 ± 3.1	41.3 ± 2.8
	DL(*n* = 24)	42.0 ± 4.0	42.0 ± 3.4	42.1 ± 4.0	41.6 ± 3.7
	DH(*n* = 28)	41.3 ± 4.4	41.7 ± 4.0	42.0 ± 3.9	41.3 ± 2.7
	P	0.660	0.603	0.673	0.921
Latency of right lower limb (ms)	C(*n* = 27)	41.1 ± 3.7	40.3 ± 3.5	40.8 ± 3.6	41.2 ± 3.5
	DL(*n* = 24)	42.2 ± 4.6	42.5 ± 5.2	42.3 ± 4.7	42.4 ± 4.5
	DH(*n* = 28)	40.1 ± 4.0	40.5 ± 4.0	40.6 ± 3.9	40.6 ± 3.4
	P	0.191	0.148	0.292	0.255

*P* > 0.05, no significant difference in the three groups. One-way analysis of variance for multiple groups of continuous variables

*Abbreviation:* *SEPs* somatosensory evoked potentials

**Table 4:** The amplitude of SEPs (P40-N45) at different points of time

		Group	Median(P25,P75)	Rank sum test	
				H	P
The amplitude of left lower limb (µV)	T1	C(*n* = 27)	0.79(0.41,1.44)	0.208	0.901
		DL(*n* = 24)	0.72(0.40,1.80)		
		DH(*n* = 28)	0.86(0.41,1.42)		
	T2	C(*n* = 27)	0.74(0.46,1.20)	0.610	0.737
		DL(*n* = 24)	0.83(0.48,1.75)		
		DH(*n* = 28)	0.84(0.47,1.72)		
	T3	C(*n* = 27)	0.86(0.50,1.25)	0.049	0.976
		DL(*n* = 24)	0.81(0.45,1.86)		
		DH(*n* = 28)	0.92(0.49,1.34)		
	T4	C(*n* = 27)	0.88(0.53,1.28)	0.172	0.917
		DL(*n* = 24)	0.72(0.46,1.72)		
		DH(*n* = 28)	0.86(0.50,1.59)		
The amplitude of right lower limb (µV)	T1	C(*n* = 27)	0.99(0.50,1.63)	0.606	0.739
		DL(*n* = 24)	1.02(0.51,1.72)		
		DH(*n* = 28)	0.83(0.48,1.41)		
	T2	C(*n* = 27)	0.83(0.50,1.52)	0.231	0.891
		DL(*n* = 24)	0.97(0.51,1.75)		
		DH(*n* = 28)	0.95(0.57,1.62)		
	T3	C(*n* = 27)	1.14(0.56,1.49)	0.007	0.996
		DL(*n* = 24)	0.96(0.54,2.09)		
		DH(*n* = 28)	0.98(0.59,1.65)		
	T4	C(*n* = 27)	0.95(0.47,1.47)	0.270	0.874
		DL(*n* = 24)	1.09(0.42,1.84)		
		DH(*n* = 28)	1.00(0.53,1.45)		

*P* > 0.05, no significant difference in the three groups. Kruskal–Wallis method for nonparametric tests

*Abbreviation:* *SEPs* somatosensory evoked potentials

### Comparison of positive/negative Cases of SEPs and TcMEPs among 3 groups

There were no statistically significant differences in the occurrence of warning SEP signals. The false-positive case rates of tcMEPs in group C and group DL were 7.4% (2/27) and 12.5% (3/24), respectively, and the false-positive case rate of tcMEPs in group DH was 42.9% (12/28). The overall mean of the three groups was statistically different (c^2^ = 11.8, *P* < 0.05). The incidence of false-positive tcMEP signals did not differ significantly between groups C and DL (*P* > 0.05), but was higher in group DH than in groups DL (*P* < 0.05) or C (*P* < 0.05). See Table 5 for more information.

**Table 5:** Comparison of positive/negative cases of SEPs and TcMEPs among 3 groups

	Group	Real positive case(%)	False positive case(%)	Real negative case(%)	False negative case(%)
SEPs	C(*n* = 27)	0(0%)	0(0%)	27(100%)	0(0%)
	DL(*n* = 24)	0(0%)	0(0%)	24(100%)	0(0%)
	DH(*n* = 28)	0(0%)	0(0%)	28(100%)	0(0%)
TcMEPs	C(*n* = 27)	0(0%)	2(7.4%)	25(92.6%)	0(0%)
	DL(*n* = 24)	0(0%)	3(12.5%)	21(87.5%)	0(0%)
	DH(*n* = 28)	0(0%)	12(42.9%)^*#^	16(57.1%)	0(0%)

*Abbreviation:* *SEPs* somatosensory evoked potentials, *TcMEPs* transcranial motor evoked potentials

^*^P < 0.05, compared with group C. Chi-square test for comparison of multiple group rates

^#^*P* < 0.05, compared with group DL. Chi-square test for comparison of multiple group rates

### Comparison of adverse reactions in 3 groups of patients

There were no significant differences in the incidence of intraoperative body movement, bradycardia, intraoperative awareness, hypotension, and postoperative nausea and vomiting between the three groups (*P* > 0.05). The incidence of hypertension differed significantly between groups DH and C (*P* = 0.017). See Table 6.

**Table 6:** Comparison of adverse reaction

Adverse reaction(number/%)	Group			
	C(*n* = 27)	DL(*n* = 24)	DH(*n* = 28)	P
Movement	0(0%)	0(0%)	0(0%)	
Intraoperative awareness	0(0%)	0(0%)	0(0%)	
Bradycardia	0(0%)	4(16.7%)	4(16.7%)	0.072
Hypertension	2(7.4%)	3(12.5%)	10(35.7%)^*^	0.017
Hypotension	6(22.2%)	7(29.2%)	7(25.0%)	0.849
Postoperative nausea and vomiting	5(18.5%)	3(12.5%)	4(14.2%)	0.857

^*^P < 0.05, compared with group C. Chi-square test for comparison of multiple group rates

## Discussion

In this randomized controlled trial of patients undergoing spine surgery, both high and low doses of Dex had little effect on SEP monitoring in intravenous inhalation combined anesthesia. In contrast, low-dose Dex had no effect on the rate of tcMEPs positive events, whereas high-dose Dex increased the rate of tcMEPs positive events significantly.

The difference between our study and previous reports is that we added a small dose of inhaled anesthetics based on propofol intravenous anesthesia, whereas previous reports studied the effect of Dex on evoked potentials under TIVA [15, 16]. TIVA has long been considered the best anesthesia method for electrophysiological monitoring of the spinal cord [17]. TIVA does not appear to be an entirely optimal anesthetic technique in spinal surgery with intraoperative SEP and tcMEP monitoring. According to a recent study, high-dose propofol during spinal surgery is associated with a significant rate of false-positive alerts on transcranial tcMEPs (also known as anesthetic fading) [18]. Meanwhile, high-dose propofol infusion (> 5 mg·kg-1·h-1) may be linked to propofol infusion syndrome after spinal surgery, which includes unexplained metabolic acidosis, ECG abnormalities, and rhabdomyolysis [19]. The addition of volatile anesthetics reduced the propofol dose during general anesthesia and, as a result, the occurrence of propofol-related side effects. Although inhaled anesthetics have been confirmed to dose-dependently inhibit evoked potentials, studies have shown that at the same concentration, desflurane has fewer effects on neural electrophysiological monitoring than sevoflurane [8]. Desflurane with a MAC of 0.3 has little effect on evoked potentials [7]. The fact that the mean propofol dosages in our trial were less than 500 mg (and less than 5 mg·kg-1·h-1) in three groups may have indicated the advantages of inhalational combination anesthesia during spinal surgery. Given the benefits of spinal ischemia–reperfusion protection and smooth recovery, it is advantageous to use Dex in patients undergoing spinal surgery [20, 21]. It is also critical to investigate how Dex affects these patients while they are under intravenous inhalational combination anesthesia. To investigate the effects of Dex dosage on evoked potentials, we created high-dose and low-dose groups based on the maximum and half of the loading and maintaining doses in the Dex administration. Our study found that the effects of dexmedetomidine at these two doses were distinct in MEP, confirming the practical significance of our findings.

In our study, the signal of SEPs could be acquired efficiently regardless of the Dex dose. Dex exerts sedative and hypnotic effects by activating the α2 receptor of locus ceruleus and stimulating endogenous sleep-inducing pathways [22]. As a result, we hypothesized that this was related to the action of Dex in the locus ceruleus without directly inhibiting the cerebral cortex. We found that, while high-dose Dex significantly increased the false positive rate of tcMEPs, low-dose Dex had no effect on tcMEPs. This meant that the effect of Dex dosage on tcMEPs should be considered in clinical application. TcMEP is the bioelectric activity recorded on the corresponding muscle or nerve surface by electrically or magnetically stimulating the motor area of the cortex or the spinal cord, depolarizing the anterior horn cells of the spinal cord or peripheral nerve motor fibers via the descending conduction pathway [23]. TcMEPs may be impacted by the muscle's own reaction capacity. Dex may influence muscular relaxation directly or indirectly by increasing the depth of sedation [24]. This could have an impact on the acquisition of tcMEPs. This could explain why the group given high doses of dexmedetomidine had a higher rate of false-positive tcMEPs. More research has to be done on the mechanism underlying Dex's effect on tcMEPs.

Similar to our findings, Lee discovered that Dex, as an adjuvant anesthetic, was more likely to result in false-positive outcomes of motor-evoked potentials during intraoperative electrophysiological monitoring of 78 adult patients in a randomized controlled trial [15]. In other spinal surgeries, however, the same conclusion was not reached. Rozet and Li discovered that the propofol-remifentanil combination with a typical clinical dose of Dex had no effect on tcMEPs and SEPs monitoring in adult patients undergoing spinal surgery [16, 25]. These studies are difficult to fully integrate due to their heterogeneity. These studies used a variety of Dex dosages, patients of varying ages, and various types of surgeries.

The changes in hemodynamics caused by Dex are notable. In the study, there was a significant difference in the incidence of hypertension between group DH and group C. Dex-related hypertension is typically transient, manifesting primarily during the loading dose, and is associated with peripheral vascular contraction [26]. With a small loading dose and a low maintenance dose, the undesirable effects of Dex could be avoided.

## Limitation

One of the study's limitations is the assessment of tcMEP responses prior to induction due to the associated pain. However, differences in data between groups suggest that high-dose Dex may increase the rate of tcMEP false positives.

## Conclusions

While propofol intravenous anesthesia was supplemented by low-concentration volatile anesthetics during spinal surgery, the use of low-dose Dex had no effect on the monitoring of SEPs and tcMEPs. High-dose Dex had no effect on SEPs monitoring, but it may make tcMEPs signals more likely to be falsely positive and cause unpleasant reactions (hypertension).

## Data Availability

The datasets used and/or analyzed during the current study are available from the corresponding author upon reasonable request.

## Abbreviations

ANOVA: Analysis of variance
ASA: American Society of Anesthesiologists
BMI: Body Mass Index
Dex: Dexmedetomidine
ECG: Electrocardiogram
Group C: Control group
Group DL: Low-dose Dexmedetomidine group
Group DH: High-dose Dexmedetomidine group
HR: Heart rate
MAC: Minimal alveolar concentration
MAP: Mean arterial pressure
SEPs: Somatosensory evoked potentials
TcMEPs: Transcranial motor evoked potentials
TIVA: Total intravenous anesthesia
TOF: Train-of-four
